# *Edwardsiella tarda* Bacteremia, Okayama, Japan, 2005–2016

**DOI:** 10.3201/eid2510.180518

**Published:** 2019-10

**Authors:** Shinya Kamiyama, Akira Kuriyama, Toru Hashimoto

**Affiliations:** Kurashiki Central Hospital, Okayama, Japan

**Keywords:** Edwardsiella tarda, bacteremia, mortality, observational study, sepsis, Japan, anaerobe, Enterobacteriaceae, antimicrobial susceptibility, bacteria, antimicrobial resistance

## Abstract

*Edwardsiella tarda* is primarily associated with gastrointestinal disease, but an increasing number of cases involving extraintestinal disease, especially *E. tarda* bacteremia, have been reported. Using clinical information of *E. tarda* bacteremia patients identified during January 2005–December 2016 in Japan, we characterized the clinical epidemiology of *E. tarda* bacteremia. A total of 182,668 sets of blood cultures were obtained during the study period; 40 (0.02%) sets from 26 patients were positive for *E. tarda*. The most common clinical manifestations were hepatobiliary infection, including cholangitis, liver abscess, and cholecystitis. Overall 30-day mortality for *E. tarda* bacteremia was 12%, and overall 90-day mortality was 27%. The incidence of *E. tarda* infection did not vary by season. We more frequently observed hepatobiliary infection in patients with *E. tarda* bacteremia than in patients with nonbacteremic *E. tarda* infections. *E. tarda* bacteremia is a rare entity that is not associated with high rates of death.

## Abstract 2

We observed more severe underlying diseases, susceptibility of isolated strains to most antimicrobial drugs, and no seasonal distribution.

*Edwardsiella tarda*, a gram-negative, facultative anaerobe that is a member of the family *Enterobacteriaceae*, typically is isolated from water environments and animals that inhabit water. It is primarily associated with gastrointestinal disease, but the number of reports of extraintestinal disease, such as septicemia, meningitis, cholecystitis, and osteomyelitis, has increased (*1*). However, little is known about the clinical epidemiology of *E. tarda* bacteremia. Therefore, we aimed to document the clinical epidemiology of *E. tarda* bacteremia, including common sources of infection, antimicrobial susceptibility, and seasonal distribution.

## Materials and Methods

We retrospectively reviewed electronic medical records and clinical microbiology records in Kurashiki Central Hospital (Okayama, Japan), a 1,166-bed, tertiary-care hospital that provides care to ≈300,000 persons annually. Clinical specimens submitted to the microbiology laboratory included blood, sputum, urine, bile, ascites, feces, placenta, tissue, and pus. Information about identified bacteria and antimicrobial susceptibility were kept as microbiology laboratory records for each specimen. We considered bacteremia to exist when >1 set of blood cultures was positive. We identified all cultures growing *E. tarda* from clinical specimens submitted during January 2005–December 2016.

We processed blood culture samples using the BacT/Alert system (Sysmex bioMérieux Co. Ltd., https://www.biomerieux.com) and conducted microbial culture using KBM Chocolate HB Agar (Kohjin Bio Co. Ltd., http://www.kohjin-bio.jp/english), KBM Sheep Blood Agar (Kohjin Bio Co. Ltd.), and BTB agar (Kyokuto Pharmaceutical Co. Ltd., https://ssl.kyokutoseiyaku.co.jp/english/index.html). We used different bacterial identification and antimicrobial susceptibility testing methods in our hospital throughout the study period. We used ID test EB-20 Nissui (Nissui Pharmaceutical Co. Ltd., https://www.nissui-pharm.co.jp/english) for bacterial identification and Kirby–Bauer disk (Eiken Chemical Co. Ltd., http://www.eiken.co.jp) for antimicrobial susceptibility testing from January 2005 through June 2007. EB-20 is a system to identify glucose-fermenting gram-negative rods by 20 patterns of biochemical properties, using hydrogen sulfide, indole, lysine, ONPG (2-nitrophenyl-β-D-galactopyranoside), adunit, inositol, rhamnose, mannit, esculin, Voges-Proskauer, arginine, urea, inositol, sorbitol, arabinose, phenylpyruvic acid, citric acid, ornithine, malonic acid, raffinose, and sugar. Thereafter, automatic systems were introduced at our hospital: DPS192 (Eiken Chemical Co. Ltd, http://www.eiken.co.jp) during July 2007–February 2013 and MicroScan WalkAway (Beckman Coulter, Inc, https://www.beckmancoulter.com/en) during March 2013–March 2014. Since April 2014, we have used MALDI Biotyper (Bruker Daltonics GmbH, https://www.bruker.com), using the manufacturer-provided database, for bacterial identification. We judged the drug susceptibility of a microorganism based on clinical breakpoints set by the Clinical and Laboratory Standards Institute; in particular, we used the document M100-S22 (*2*) during June 1, 2013–December 31, 2016.

We collected all clinical information of patients with positive *E. tarda* bacteremia results from electronic medical records, including age, sex, underlying diseases, source of infection, antimicrobial drug administered, treatment period, and outcome. We defined chronic kidney disease as a serum creatinine level of >2.0 mg/dL (reference range 0.65–1.07 mg/dL) and chronic liver disease as liver cirrhosis or chronic hepatitis B or C infection. We defined nosocomial bloodstream infection, healthcare-associated bloodstream infection, community-acquired bloodstream infection, and febrile neutropenia according to the previous study and guideline (*3*,*4*). We defined 30-day mortality as patient death within 30 days after the onset of *E. tarda* bacteremia and 90-day mortality as patient death within 90 days after onset. We also collected information of patients with *E. tarda* nonbacteremic infections.

We described the clinical characteristics and 30-day mortality of patients with *E. tarda* bacteremia, along with the source of infection and antimicrobial susceptibility. We then compared the characteristics of patients with *E. tarda* bacteremia by 30-day mortality. We also compared the characteristics of patients with bacteremic and nonbacteremic *E. tarda* infections. We also conducted an exploratory multivariable logistic regression analysis to investigate the risk for *E. tarda* bacteremia incidence among all *E. tarda* infections.

Because a previous literature review suggested seasonal variation in the occurrence of *E. tarda* bacteremia (*5*), we thus examined whether such variation or trend existed in the cases in our study by using Cochran-Armitage test. We tested dichotomous variables with Fisher exact test and and continuous variables by Wilcoxon signed-rank test. Statistical analysis was performed using Stata version 15.1 (StataCorp, http://www.stata.com). We considered p<0.05 to be statistically significant.

The Ethics Committee of Kurashiki Central Hospital approved this study (no. 2,527). Only persons with appropriate authorization had access to participants’ records, and patient confidentiality was maintained. Given the nature of a retrospective chart review, written consent from the patients was waived.

## Results

We obtained 182,668 sets of blood cultures during the study period, of which 19,234 sets were positive for some organisms and 40 sets from 26 patients were *E. tarda*–positive. *E. tarda* bacteremia was diagnosed in 26 patients (13 men and 13 women); their median age was 75 years (range 45–101 years) (Table 1).

**Table 1:** Characteristics of patients with *Edwardsiella tarda* bacteremia, Kurashiki Central Hospital, Okayama, Japan, 2005–2016*

Characteristic	Total, N = 26	Survivors, n = 23	Patients who died within 30 d after bacteremia onset, n = 3	p value
Median age, y (IQR) [range]	75 (63–85) [45–101]	75 (64–85) [45–101]	63 (30–87) [60–87]	0.55
Sex, no. patients				
M	13	11	2	1.00
F	13	12	1	
Underlying disease, no. patients				
Solid tumor	12	10	2	0.58
Cardiovascular disease	4	4	0	1.00
Diabetes mellitus	3	3	0	1.00
Gallstone	3	3	0	1.00
Chronic liver disease	2	1	1	0.22
Cerebrovascular disease	2	2	0	1.00
Hematologic malignancy	1	1	0	1.00
Chronic kidney disease	0	0	0	NE
Ulcerative colitis	0	0	0	NE
Crohn disease	0	0	0	NE
None	4	2	2	0.052
Other	0	0	0	NE
Behavioral/dietary risk factors, no. patients				
Alcoholism	4	2	2	0.052
Exposure to raw food	3	3	0	1.00
Exposure to fresh or marine water, animal feces	1	1	0	1.00
Clinical diagnosis, no. patients				
Cholangitis	9	9	0	0.53
Liver abscess	6	6	0	1.00
Enterocolitis	4	4	0	1.00
Cholecystitis	3	3	0	1.00
Spontaneous bacterial peritonitis	1	0	1	0.115
Mycotic aneurysm	1	1	0	1.00
Necrotizing fasciitis	1	0	1	0.115
Empyema	1	0	1	0.115
Febrile neutropenia	1	1	0	1.00
Osteomyelitis	1	1	0	1.00
Secondary peritonitis	1	1	0	1.00
Focus unknown	5	4	1	0.49
Receipt of chemotherapy for cancer	4	4	0	1.00
Median duration of treatment for infection, d (IQR) [range]†	12 (7–27) [1–77]	13 (8–30) [1–77]	5 (2–11) [2–11]	0.084

*IQR, interquartile range; NE, not evaluated.

### Clinical Characteristics

Some patients had >1 underlying disease: solid tumors (12 patients), cardiovascular diseases (4 patients), diabetes mellitus (3 patients), gallstone disease (3 patients), chronic liver disease (2 patients), cerebrovascular disease (2 patients), and hematologic malignancy (1 patient) (Table 1). Four patients had no underlying disease. Sites of solid tumors included pancreas (3 patients), gallbladder/bile duct (3 patients), colon (2 patients), and esophagus, gastric, liver, and thyroid (1 patient each). Of the 12 patients with solid tumors, 4 were receiving chemotherapy for their cancer when *E. tarda* bacteremia occurred.

Clinical diagnoses by the site of infection were cholangitis (9 patients); liver abscess (6 patients); enterocolitis (4 patients); cholecystitis (3 patients); and spontaneous bacterial peritonitis, mycotic aneurysm, necrotizing fasciitis, empyema, osteomyelitis, and secondary peritonitis (1 patient each) (Table 2). Seventeen patients had community-acquired bloodstream infections. The source of infection was not identified in 5 patients, including 1 with febrile neutropenia; 3 patients had nosocomial bloodstream infections, and 6 had healthcare-associated bloodstream infections.

**Table 2:** Clinical characteristics of 26 patients with *Edwardsiella tarda* bacteremia, Kurashiki Central Hospital, Okayama, Japan, 2005–2016*

Patient no.	Age, y/sex	Clinical diagnosis	Underlying disease	Treatment	Treatment duration, d	Concurrent organisms (source)	Outcome
1	77/M	Focus unknown	Cerebrovascular disease	LVX	3	*E. coli*, *B. fragilis* (pus)	Recovered
2	79/M	Liver abscess	Cardiovascular disease	CFP/SUL→IPM/CIL→PIP→MEP, CLI→MEM	38		Recovered
3	70/F	Cholangitis, cholecystitis	None	CFP/SUL	8		Recovered
4	87/M	Focus unknown	Hepatocellular carcinoma	FEP	11		Died at 12 d
5	62/M	Mycotic aneurysm, liver abscess, osteomyelitis	Diabetes mellitus	IPM/CIL→AMP, GEN→SAM→VCM, PNP→VCS→PZX	30		Died at 39 d
6	92/F	Focus unknown	Colon cancer	CRO	30		Died at 32 d
7	89/F	Focus unknown	Colon cancer	CRO→MEM	16		Recovered
8	85/F	Liver abscess, enterocolitis	Thyroid cancer	MEM, CLI→MEM→IPM/CIL	21		Recovered
9	88/F	Cholangitis	Cholangiocarcinoma	IPM/CIL	7		Died at 40 d
10	75/F	Cholecystitis	None	PZX	3	*Klebsiella* sp., *E. coli* (bile)	Recovered
11	101/F	Cholangitis	None	CFP/SUL	10		Recovered
12	61/M	Enterocolitis	Cardiovascular disease	CRO→LVX	10		Recovered
13	58/M	Liver abscess	Gallbladder cancer、 invasion of liver	CFP/SUL→MEM→MIN→AMP, MIN	77	*E. faecalis*, *E. faecium*, *C. freundii*, *Bacteroides* sp. (pus)	Recovered
14	84/F	Cholangitis	Cardiovascular disease, cerebrovascular disease	CRO	6		Recovered
15	83/F	Cholangitis	Pancreatic cancer	CFP/SUL	1		Recovered
16	66/M	Liver abscess	Pancreatic cancer	CFZ→LEX	46		Recovered
17	85/M	Enterocolitis	Cardiovascular disease	CRO→LVX	12		Recovered
18	64/F	Enterocolitis	Chronic liver disease, diabetes mellitus	CMZ→AMP	13		Recovered
19	74/F	Secondary peritonitis	Diabetes mellitus	CMZ→TZP	27	*E. coli*, *K. pneumoniae*, *S. anginosus*, *F. nucleatum* (ascites)	Recovered
20	63/M	Necrotizing fasciitis	None	MEM, CLI	2		Died at 2 d
21	45/F	Liver abscess, cholangitis	Pancreatic cancer	CFP/SUL→AMP	31	*S. anginosus* (blood)	Died at 45 d
22	65/M	Cholangitis, cholecystitis	Gastric cancer, gallstone	CFP/SUL→AMP	13	*S. gallolyticus* (blood)	Recovered
23	81/M	Cholangitis	Gallstone, esophageal cancer	CFP/SUL→AMP	16		Recovered
24	64/M	Cholangitis	Cholangiocarcinoma, gallstone	CFP/SUL→AMP	8		Recovered
25	59/M	Focus unknown, febrile neutropenia	Peripheral T-cell lymphoma	CZO	12		Recovered
26	60/F	Spontaneous bacterial peritonitis, empyema	Chronic liver disease	TZP→AMP	5		Died at 6 d

*AMP, ampicillin; *B. fragilis*, *Bacteroides fragilis*; CFP/SUL, cefoperazon–sulbactam; *C. freundii*, *Citrobacter freundii*; CFZ, cefazolin; CLI, clindamycin; CMZ, cefmetazole; CRO, ceftriaxone; CZO, cefozopran; *E. faecalis*, *Enterococcus faecalis*; *E. faecium*, *Enterococcus faecium*; *E. coli*, *Escherichia coli*; FEP, cefepime; *F. nucleatum*, *Fusobacterium nucleatum*; GEN, gentamycin; IPM/CIL, imipenem–cilastatin; *K. pneumoniae*, *Klebsiella pneumoniae*; LEX, cephalexin; LVX, levofloxacin; MEM, meropenem; MIN, minocycline; PNP, panipenem; PIP, piperacillin; PZX, pazufloxacin, SAM, ampicillin sulbactam; *S. anginosus*, *Streptococcus anginosus*; *S. gallolyticus*, *Streptococcus gallolyticus*; Tx, treatment; TZP, piperacillin tazobactam. Arrows indicate the order of antimicrobial drugs used. Blank cells indicate no other concurrent organisms.

Patients with *E. tarda* bacteremia were older and more likely to have solid tumors than were patients with *E. tarda* nonbacteremic infections (Table 3). In addition, we observed hepatobiliary infection, such as cholangitis and liver abscess, more frequently in patients with bacteremia.

**Table 3:** Comparison of characteristics of patients with bacteremic and nonbacteremic *Edwardsiella tarda* infection, Kurashiki Central Hospital, Okayama, Japan, 2005–2016*

Patient characteristic	Patients with bacteremic infection, n = 26	Patients with nonbacteremic infection, n = 124	p value
Median age, y (IQR) [range]	75 (63–85) [45–101]	56 (12–73) [0- 89]	<0.001
Sex, no. patients			
M	13	82	0.178
F	13	42	
Underlying disease, no. patients			
Solid tumor	12	22	0.004
Cardiovascular disease	4	22	1.00
Diabetes mellitus	3	13	1.00
Gallstone	3	13	1.00
Chronic liver disease	2	7	0.65
Cerebrovascular disease	2	1	0.078
Hematologic malignancy	1	1	0.32
Chronic kidney disease	0	1	1.00
Ulcerative colitis	0	14	0.13
Crohn disease	0	1	1.00
None	4	52	0.013
Other	0	2	1.00
Behavioral/dietary risk factors, no. patients			
Alcoholism	4	9	0.24
Exposure to raw food	3	7	0.38
Exposure to fresh or marine water, animal feces	1	0	0.173
Clinical diagnosis, no. patients			
Cholangitis	9	8	<0.001
Liver abscess	6	1	<0.001
Enterocolitis	4	74	0.076
Cholecystitis	3	9	0.44
Spontaneous bacterial peritonitis	1	0	0.173
Mycotic aneurysm	1	1	0.32
Necrotizing fasciitis	1	0	0.173
Empyema	1	0	0.173
Febrile neutropenia	1	0	0.173
Osteomyelitis	1	0	0.173
Secondary peritonitis	1	0	0.173
Focus unknown	5	1	0.001
Endometriosis	0	1	1.00
Appendicitis	0	4	1.00
Congenital infection	0	1	1.00
Cystitis	0	1	1.00
Intraabdominal abscess	0	1	1.00
Perianal abscess	0	1	1.00
Pneumonia	0	2	1.00
Pyometra	0	1	1.00
Secondary peritonitis	0	2	1.00
Superficial surgical site infection	0	1	1.00
Receipt of chemotherapy for cancer, no. patients	4	7	0.099
Median duration of treatment for infection, d (IQR) [range]	12 (7–27) [1–77]	5 (3–9) [0–36]	<0.001

*IQR, interquartile range.

Because the cohort included 26 *E. tarda* bacteremia patients, we conducted a multivariable logistic regression analysis adjusted with 2 explanatory variables. We hypothesized that underlying liver disease and old age could be associated with the incidence of *E. tarda* bacteremia and selected these 2 variables as the covariates. Our analysis suggested that age >65 years was significantly associated with an increased risk for *E. tarda* bacteremia incidence (odds ratio 2.70; 95% CI 1.11–6.55; p = 0.028). However, underlying chronic liver disease was not the risk factor for *E. tarda* bacteremia (odds ratio 2.48; 95% CI 0.41–14.99; p = 0.32).

### Treatment and Outcomes

All *E. tarda* strains isolated from blood cultures were susceptible to all tested antimicrobial drugs. *E. tarda* bacteremia patients were treated with a variety of antimicrobial drugs according to the treating physicians’ discretion (Table 3). The median duration of treatment was 12 days (range 1–77 days). Overall 30-day mortality for *E. tarda* bacteremia was 12% (3/26) and overall 90-day mortality 27% (7/26).

Patient 4 had end-stage hepatocellular carcinoma and liver failure. On day 2 after admission, *E. tarda* bacteremia developed; the source of infection was unidentified. He was treated with cefepime and promptly became afebrile. *E. tarda* bacteremia was considered controlled by cefepime; however, the patient died of hepatic failure on day 11.

In patient 20, necrotizing fasciitis was diagnosed, and *E. tarda* was detected from wound and blood cultures. Although meropenem and clindamycin were administered, he died on day 2.

Patient 26, who had end-stage alcoholic liver cirrhosis, was admitted for massive pleural effusion and ascites. *E. tarda* was detected from pleural effusion but not from ascites. Empyema and spontaneous bacterial peritonitis caused by *E. tarda* were diagnosed. Although these fluids were drained and antimicrobial drugs were given, she died on day 5.

Patient 5 was admitted for evaluation of fever and back pain. Blood cultures drawn on admission day revealed *E. tarda*, and he was treated with imipenem–cilastatin. However, his fever persisted. Computed tomography scan of the chest and abdomen revealed mycotic thoracic aneurysm, liver abscess, and vertebral osteomyelitis. He was treated with multiple antimicrobial drugs but died of a ruptured mycotic aneurysm on day 39.

In patients 6, 9, and 21, *E. tarda* bacteremia developed and improved with antimicrobial therapy. However, these patients died of underlying diseases.

### Seasonal Variation in *E. tarda* Bacteremia

The incidence of *E. tarda* infection did not vary by season (Figure). We found no trend of *E. tarda* bacteremia incidence among all *E. tarda *infections when we examined them by month (p = 0.46) or by season, defined as a set of 3 months (p = 0.53).

**Figure:**
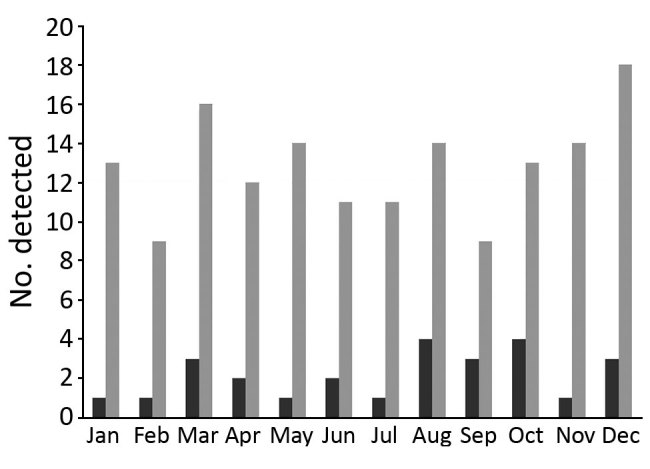
Seasonal variation in the incidence of *Edwardsiella tarda* infection, Kurashiki Central Hospital, Okayama, Japan, 2005–2016. Black bars, blood culture; gray bars, all specimens (including blood cultures).

## Discussion

*E. tarda* is associated with freshwater and marine life, including fish, reptiles, and amphibians (*1*). The organism resembles *Salmonella* biochemically and clinically (*1*). *Salmonella* usually ferments D-mannitol, urease, oxidase, and D-sorbitol, whereas *E. tarda* produces hydrogen sulfide and indole (*6*).

*E. tarda* is a rare human pathogen and is primarily associated with gastrointestinal diseases, including the asymptomatic carrier state (*1*). Approximately 80% of infections are intestinal. *E. tarda* causes a *Salmonella*-like gastrointestinal infection, usually self-limited enteritis, with intermittent watery diarrhea and low-grade fever (*1*,*7*).

The pathogenesis of *E. tarda* and its disease-causing mechanism remain unclear. Twelve classes of bacterial protein secretion systems are known; these systems transport virulence proteins into the cell and, in some cases, directly into the cytoplasm of a target cell (*8*). The bacterial type III and type VI secretion systems (T3SS and T6SS) are believed to play an essential role in *E. tarda* survival, replication, and virulence inside the host. In particular, T6SS is proposed to enable *E. tarda* to establish inside the host, cause severe systemic infection, and eventually kill the host.

We reviewed 26 cases of *E. tarda* bacteremia. Clinical diagnoses included 15 (58%) biliary tract infections (cholangitis, cholecystitis, and liver abscess). Eight of these patients had hepatobiliary diseases including cholangiocarcinoma, gallbladder cancer, pancreatic cancer, gallstone disease. Therefore, hepatobiliary diseases may be a predisposing factor of *E. tarda* biliary tract infections. However, our multivariable logistic regression found that only age >65 years was associated with the incidence of *E. tarda* bacteremia. We acknowledge that the sample size of our study and the number of *E. tarda* bacteremia incidence were still small, and thus the finding from our multivariable analysis might be only exploratory.

Previous studies reported high rates of death for *E. tarda* bacteremia, ranging from 22.7% to 44.6% (*1*,*5*,*9*). In contrast, the death rate for patients with *E. tarda* bacteremia in the cohort reported here was low at 12%. However, 2 of these 3 patients had end-stage liver disease; only 1 death among these patients was attributed to *E. tarda* bacteremia.

*E. tarda* is susceptible to most antimicrobial drugs, including tetracyclines, aminoglycosides, quinolones, antifolates, chloramphenicol, nitrofurantoin, fosfomycin, and most β-lactams (*10*), and is naturally resistant to benzylpenicillin, colistin, and polymyxin B (*1*,*11*). In our study, *E. tarda* was susceptible to most commonly used antimicrobial drugs. *E. tarda* susceptibilities to colistin and polymyxin B are unknown because susceptibility testing is not routinely performed for these drugs in our institution. Previous studies have shown that all strains of *E. tarda* were positive for β-lactamase production examined with nitrocefin β-lactamase disks, but an ampicillin-resistant *E. tarda* strain has not been reported (*10*,*11*). Whether *E. tarda* isolates detected in our institution produced β-lactamase is not clear because we did not perform the β-lactamase test, but 5 cases were successfully treated with ampicillin.

Hirai et al. suggested that *E. tarda* bacteremia is likely to develop during summer and autumn months in the Northern Hemisphere (*8*). The authors conducted a literature review of 77 *E. tarda* bacteremia cases reported from diverse areas and suggested seasonal variation in incidence for 22 cases. Our study of 26 *E. tarda* bacteremia cases suggests no such seasonal distribution. Several possible reasons might account for this discrepancy. First, *E. tarda* can colonize. In our study, hepatobiliary infection (such as cholangitis, cholecystitis, and liver abscess) was diagnosed in 58% (15/26) patients, and patients colonizing *E. tarda* developed *E. tarda* bacteremia. Second, diversity might exist in the patients’ dietary patterns.* E. tarda* frequently infects fish. Hirai et al. included patients from many parts of the world, so the intake of fish might have differed according to the season or geographic area across reports. In contrast, our study included only people in a single area of Japan who habitually ate raw seafood, such as sashimi, throughout the year; this tendency might have led to no seasonal variation of *E. tarda* bacteremia incidence. Third, our study had no missing clinical data for any patients, whereas Hirai et al. examined 22 of all 77 eligible patients, which might have rendered their analysis vulnerable to information bias. 

Our study had some strengths. First, we elucidated that no seasonal variation existed in *E. tarda* bacteremia in this population. Second, we described the characteristics of each patient with *E. tarda* bacteremia and provided risk factors for *E. tarda* bacteremia incidence among all *E. tarda* infections.

Our study also had some limitations. First, the number of blood cultures submitted increased in recent years in our hospital. The number of blood cultures submitted in 2016 nearly doubled that for 2005. This increase might have resulted in the underestimation of *E. tarda* bacteremia in the earlier years of our study period. Second, ours was a retrospective and single-center study. However, our study had no missing data regarding clinical information. Furthermore, we successfully presented a particularly large case series of *E. tarda* bacteremia.

In conclusion, *E. tarda* bacteremia is a rare disease that is not associated with high rates of death. *E. tarda* bacteremia patients in our cohort in Japan had more severe underlying diseases, such as hepatobiliary disease and solid tumors, than did patients in previous studies. Hepatobiliary infections, such as cholangitis, cholecystitis, and liver abscess, are the most common clinical manifestations in patients with *E. tarda* bacteremia. The major underlying diseases in this study were hepatobiliary diseases and malignancy. Furthermore, *E. tarda* strains we isolated were susceptible to most antimicrobial drugs, including β-lactams, aminoglycoside, tetracycline, fosfomycin, fluoroquinolone, and trimethoprim/sulfamethoxazole, and *E. tarda* bacteremia was successfully treated with ampicillin. Finally, we observed no seasonal distribution of *E. tarda* bacteremia. Risk factors for *E. tarda* bacteremia–related death remain to be investigated.
